# Using transcriptome analysis to evaluate the impact of *dsAllim* cotton on non-target organism *O. similis*

**DOI:** 10.3389/fpls.2025.1720420

**Published:** 2026-01-27

**Authors:** Changyan Li, Haiqin Yao, Kunwei Hua, Danyang Cao, Hang Zhang, Desuo Yin, Xiaolian Zhang, Feng Wang, Weihua Ma, Lizhen Chen, Aiqing You

**Affiliations:** 1 Food Crops Institute, Hubei Academy of Agricultural Sciences, Hubei Key Laboratory of Food Crop Germplasm and Genetic Improvement, Laboratory of Crop Molecular Breeding, Ministry of Agriculture and Rural Affairs, Wuhan, Hubei, China; 2 College of Food Science and Technology, Wuhan Business University, Wuhan, Hubei, China; 3 Hubei Insect Resources Utilization and Sustainable Pest Management Key Laboratory, College of Plant Science and Technology, Huazhong Agricultural University, Wuhan, Hubei, China; 4 Guizhou Academy of Tobacco Science, Tobacco Molecular Genetics Key Laboratory of China Tobacco, Guiyang, Guizhou, China

**Keywords:** biosafety, double-stranded RNA, genetically modified crop, natural enemy, transcriptomic entropy

## Abstract

**Introduction:**

The application of genetically engineered (GE) crops in pest management raises biosafety concerns among governments, the scientific community, and the public, especially with the emergence of RNA interference (RNAi)-based crops expressing insecticidal double-stranded RNA (dsRNA). These crops may pose challenges to public health, agriculture, and conservation, and they could also present risks to non-target organisms, including beneficial natural enemies of pests. Natural enemies of insects are a significant component of global biodiversity and play a crucial role in managing insect pests within agroecosystems. This study addresses the biosafety concerns associated with insect-resistant transgenic dsRNA-expressing crops, focusing on their potential unintended effects on non-target organisms, particularly natural enemies.

**Methods:**

We combined biological and bioinformatic approaches, utilizing both food-chain delivery and animal-feeding systems, to comprehensively evaluate the potential unintended effects of exogenous insecticidal dsRNA expressed by dsAllim cotton on the biological parameters and transcriptome of the cotton-field predatory natural enemy, *Orius similis*.

**Results:**

The findings indicate that dsAllim cotton had no adverse effects on *O. similis*, suggesting its potential safety for non-target beneficial insects. At both developmental and transcriptomic levels, ds*Allim* cotton showed no significant impact on *O. similis*.

**Discussion:**

These results support the use of ds*Allim* cotton as a reference in developing regulatory frameworks for the risk assessment of RNAi crops. Together with previous research, our findings underscore the importance of conducting RNAi crop safety evaluations for non-target organisms on a case-by-case basis, with particular attention to potential off-target effects.

## 1 Introduction

Insect-resistant genetically engineered (IRGE) crops that use RNA interference (RNAi), so-called RNAi crops, such as the corn rootworm-resistant maize, have been successfully commercialized and released onto the market. However, the impact of RNAi crops on the environment also requires in-depth research to help us better understand their potential risks. As a modern new breeding method, genetically modified crop breeding can accurately change the genetic composition of target crops compared to hybrid breeding, mutagenesis breeding and other breeding methods, making biological traits more in line with human needs. In order to continuously adapt to the needs of emerging RNAi crop applications and research and development, the current safety assessment procedures for genetically modified organisms still need to be improved and perfected (Heinemann et al., 2013; National Academies of Sciences, Engineering, and Medicine, 2016; Heinemann, 2019). However, a standard framework for environmental risk assessment of RNAi crops in China is absent so far.

RNAi is an evolutionarily conserved post-transcriptional gene silencing (PTGS) phenomenon in which small RNAs inhibit target-gene expressions or translations by degrading targeted mRNAs (Tomari and Zamore, 2005; Ghildiyal and Zamore, 2009; Cooper et al., 2019; Zhao and Guo, 2022). RNAi is rapidly becoming a modern approach to the management of important agricultural pests due to its green, efficient and target-specific characteristics (Carstens et al., 2012; Kupferschmidt, 2013; Smagghe and Swevers, 2014; Niu et al., 2021). RNAi crops achieve insect-pest control by specifically expressing double-stranded (ds) RNA that cause reduced expression of insect essential genes after they feed on RNAi crops, which then affect their growth or development and even lead to death (Borges and Martienssen, 2015; Li et al., 2020; Papadopoulou et al., 2020).

Similar to other IRGE products, most countries require RNAi crops to undergo a rigorous assessment before they can be approved for environmental release (Whangbo and Hunter, 2008; Niu et al., 2021). The assessment of potential unintended effects on non-target organisms (NTOs) are an important part of such environmental risk assessments. Although the insecticidal dsRNA in RNAi crops is specifically selected, studies have shown that RNAi often produces off-target effects on non-target genes. But for current off target research, the sequence similarity does not itself guarantee a significant phenotypic effect in crops or insert by the primary dsRNA, in silico screening may help to identify appropriate experimental endpoints within a risk assessment framework for pesticidal RNAi (Jackson et al., 2003; Fedorov et al., 2006; Mogren and Lundgren, 2017), which results in unintended consequences in NTOs exposed to RNAi crops (Lundgren and Duan, 2013).

Insects’ natural enemies form part of global biodiversity, which are also of great importance in insect-pest management, in agroecosystems (Segoli et al., 2023). Generally, assessing the potential unintended effects of IRGE crops on predatory natural enemies can be done using laboratory bioassays, the insecticidal elements could be delivered by food-chain or mixed with artificial diet (Li et al., 2017). Laboratory bioassays typically focus on insect life history parameters. In animal-feeding tests, a maximum hazard dose of the insecticidal factors (for example, >10× dosage of the field exposure concentration by directly feeding synthesized dsRNA) are expressed by the IRGE crops, to amplify the adverse effects (Romeis et al., 2008; Chen et al., 2021). Considering the inefficient prediction of off-target in RNAi crops, application of next-generation sequencing and appropriate bioinformatic tools are key to determine the molecular changes that occur at the whole transcriptome level (Good et al., 2016; Allen, 2017; Arpaia et al., 2020). To determine the distinct molecular processes among NTO species, some case-by-case evaluation of the safety of RNAi crops has been reported (San Miguel and Scott, 2016; Ni et al., 2017; Wu et al., 2022).

The term “LIM” is an acronym derived from the names of three proteins: Lin-11, Isl-1, and Mec-3 (Ostendorff et al., 2002). It represents a family of cysteine-rich proteins that possess one or more zinc finger structures. All LIM protein molecules contain an LIM domain, which is defined by a specific conserved sequence, CX_2_CX_16–23_HX_2_CX_2_CX_2_CX_16–21_CX_2_(C/H/D) (Sanchezgarcia and Rabbitts, 1994). The transgenic ds*Allim* cotton has been developed to confer resistance against *Apolygus lucorum*, a hemipteran insect-pest. The expression of the *Allim* gene in *A. lucorum* larvae which ingest ds*Allim* cotton is suppressed. This affects muscle development and death as a result of failed molting (Liang et al., 2021). The *O*. *similis* is a dominant predatory natural enemy in cotton fields. Both larvae and adults consume phytophagous insect-pests, but also cotton pollen, which serve as the potential exposure route to GE cotton (Wang et al., 2018). In this study, we chose ds*Allim* cotton and *O. similis* as representative subjects for a comprehensive evaluation of the safety of RNAi crops. The evaluation framework performed in this study encompassed the identification of exposure routes, the execution of food-chain bioassays, direct-feeding tests, off-target effect analyses, and transcriptomic profiling. These methodologies are consistent with international safety assessment standards. According to the guidance provided by the European Food Safety Authority (EFSA) for genetically modified plants (GMPs), particularly those developed through RNAi, applicants are mandated to conduct additional analyses. These include: (1) bioinformatic analysis to identify potential off-target gene silencing effects; (2) case-by-case evaluations beyond basic insertion and expression data, tailored to the specific nature of the introduced trait; (3) assessment of RNA stability, with particular attention to the persistence of non-coding RNA in the digestive system and its potential impacts on humans and animals; and (4) precise estimation of dietary exposure to evaluate the intake level of transgenic components under typical dietary conditions (EFSA et al., 2025). This comprehensive approach reflects the current understanding of RNAi mechanisms and supports the development of science-based regulatory frameworks. Our findings provide valuable data to support future research and regulatory assessments of RNAi crops.

## 2 Materials and methods

### 2.1 Insect rearing and plant materials

*O*. *similis* were initially collected from cotton fields in Wuhan (Hubei Province, China) and reared on *Aphis craccivora* in an incubator (26 ± 2 °C temperature, 75 ± 5% relative humidity and 14 h L: 10 h D photoperiod). The *A. craccivora* were provided by Dr. Xingmiao Zhou from Huazhong Agricultural University (Wuhan, Hubei Province) and were propagated for several generations on *Vicia faba* in a growth chamber (25 ± 1 °C temperature, 70 ± 5% relative humidity and 14 h L: 10 h D photoperiod).

The study used the ds*Allim* cotton line and its corresponding non-transformed receptor cultivar as plant materials. The full length of *AlLIM* cDNA was isolated from *A. lucorum* and a conserved domain of 403 bp was chosen as the target sequence for RNAi in cotton, which passed the safety check by homologous examination against other insects and Human genomic or transcript libraries. The ds*Allim* was driven by *Agrobacterium*-mediated genetic transformation with the pHellsgate4 RNAi vector and has high resistance to *A. lucorum* (Liang et al., 2021). Both cotton lines were generously provided by Shuangxia Jin (National Key Laboratory of Crop Genetic Improvement, Huazhong Agricultural University, Wuhan, China). Cotton seedlings were planted on experimental plots (10.5-m long, 4.5-m wide) in Huazhong Agricultural University. During the planting period, no pesticide was applied.

### 2.2 Synthesis of dsRNAs

Pairwise sequence alignment was carried out to obtain the *Oslim* gene sequence most homogenous to the *Allim* sequence, from our local *O. similis* transcriptome (Liang et al., 2021). Subsequently, ds*Allim*, ds*Oslim* and ds*GFP* (Zhou et al., 2023) were amplified by PCR using primers containing the T7 promoter (**Supplementary Table S1**). The dsRNAs were synthetized using a dsRNA Synthesis Kit (Thermo Fisher Scientific, USA).

### 2.3 Food-chain delivery bioassay

At the four-leaf stage of both transgenic and non-transgenic cotton seedlings, the *A. craccivora* were transferred and reared on these plants for 3 generations. The aphids were used to feed *O. similis*. One 2^nd^ instar *O. similis* nymph was introduced to a fresh cotton leaf and aphid nymphs in a Petri dish (Φ = 90 mm). Insect life history was then recorded. Each treatment had 3 replicates, and each replicate consisted of 20 *O. similis* nymphs. The methods and conditions for aphid breeding refer to our previously published related papers (Liang et al., 2021).

### 2.4 *O. similis*-feeding test

An artificial diet has been reported in 2008, which were no significant differences in the period of pre-oviposition, the fecundity and longevity of the adult of *O. similis* between the treatments and the control (Zhang et al., 2008). One newly molted (< 24 h) 4^th^ instar nymph of *O. similis* was introduced to artificial diet mixed with dsRNAs (ds*Allim*, ds*Oslim* and ds*GFP*) or ddH_2_O in one Petri dish. The ds*Oslim* served as positive control, while the ds*GFP* served as the negative control. The concentrations of each dsRNA were 100× higher than that expressed in cotton leaves of the ds*Allim* cotton (4.4 ng/g) (Liang et al., 2021). The artificial diet was changed daily. Ten newly emerged adults (female: male = 6: 4) were placed in a cylinder (Φ = 12 cm, 10 cm high) supplied with *A. craccivora*. Then 3–4 branches of *Jasminum mesnyi* were placed in each cylinder to serve as egg-laying hosts for *O. similis*. Insect life history was then recorded. Each treatment had 3 replicates, and each replicate consisted of 20 *O. similis* adults.

### 2.5 Uptake efficiency of dsRNA in *O. similis*-feeding test

To evaluate the uptake efficiency of dsRNA in the feeding test system, newly molted 3^rd^ instar nymphs and adults of *O. similis* were used. The dsRNAs (ds*Allim*, ds*Oslim* and ds*GFP*) were mixed with artificial diet as described above. The nymph and adult samples were collected at 4 or 7 d, and 4 or 8 d, respectively, after which qRT-PCR was performed to measure RNAi efficiency. For transcriptome sequencing, samples were collected from both the ds*GFP* (control) and ds*AlLIM* (treatment) groups at two time points: nymphs fed for 6 days and adults fed for 7 days.

### 2.6 Quantitative Real-Time PCR

Following the manufacturer’s instructions, total RNA was extracted with the RNAiso reagent (Takara, Kyoto, Japan). cDNA was prepared with a reverse transcription kit (Takara, Kyoto, Japan). The 10 μl qRT-PCR reaction mixture consisted of 5 µl of SYBR solution (Takara, Kyoto, Japan), 0.8 µl of specific primers (**Supplementary Table S2**), 2.2 µl of double distilled water, 2 µl of cDNA. The reaction mixture was placed in a 96-well Microseal plate. The qRT-PCR was performed on Bio-Rad Detection iQ2 System (Bio-Rad, Hercules, CA, USA). The qRT-PCR program was 95 °C for 30 s, followed by 40 cycles of 95 °C for 5 s and 60 °C for 30 s. The *O. similis β-actin* gene was used as an endogenous reference gene for the run. The 2^-ΔΔCt^ method was used to calculate the relative transcript levels of the corresponding genes (Livak and Schmittgen, 2001).

### 2.7 Off-target effects and transcriptome analysis

The samples collected above were also used for RNA-Seq on an Illumina platform in MetWare (Wuhan, China). The raw reads were filtered, and the clean reads were *de novo* assembled into unigenes using Trinity. Transcript abundance was then estimated using RSEM. Differential expression analysis was performed using DESeq2. The screening of differentially expressed genes (DEGs) was combined with log_2_ (Fold Change) and false discovery rate (FDR). Genes with |log_2_ (Fold Change) | > 1 and FDR < 0.05 were considered as DEGs. The enrichment analysis is performed based on the hypergeometric test. For KEGG, the hypergeometric distribution test is performed with the unit of pathway; for GO, it is performed based on the GO term. Several randomly selected DEGs from each group were validated using qRT-PCR. The off-target effects of dsRNA were assessed by the correlation between the number of base-pairs matched and their fold changes for each gene containing continuous matched base-pairs using Perl scripts. Furthermore, another 2 non-target genes were also considered. The first one was the homolog genes of the target genes, which were identified using BLASTN (E-value < 1E-10). The second one was genes in the same pathway as the target genes. Besides, the transcriptome was evaluated using the Shannon entropy count, which was calculated using R script. Defining transcriptomic changes as Shannon entropy allowed transcriptome variation to be displayed as a separate metric (Martínez and Reyes-Valdés, 2008). The bioinformatic analysis was adopted from our previous study Wu et al., 2022).

### 2.8 Statistical analysis

SPSS 26.0 was used for the statistical analysis of the data. Data of the biological assays were analyzed using one-way ANOVA, and data produced by qRT-PCR were analyzed using Student’s t test.

## 3 Results

### 3.1 *O. similis* show risk of field exposure to ds*Allim*

The ds*Allim* content in aphis after feeding on ds*Allim* cotton leaves for 48 h, was (29.0 ± 1.4) × 10–^2^ ng g^-1^. The ds*Allim* content in *O. similis* when it fed on aphis that had fed on ds*Allim* cotton leaves for 48 h, was14.0 ± 1.6 × 10–^4^ ng g^-1^. This showed that *O. similis* was at high probability from indirect exposure to ds*Allim* in the field.

### 3.2 Life-table parameters of *O. similis* were not affected through the food-chain delivery system

**Figure 1:**
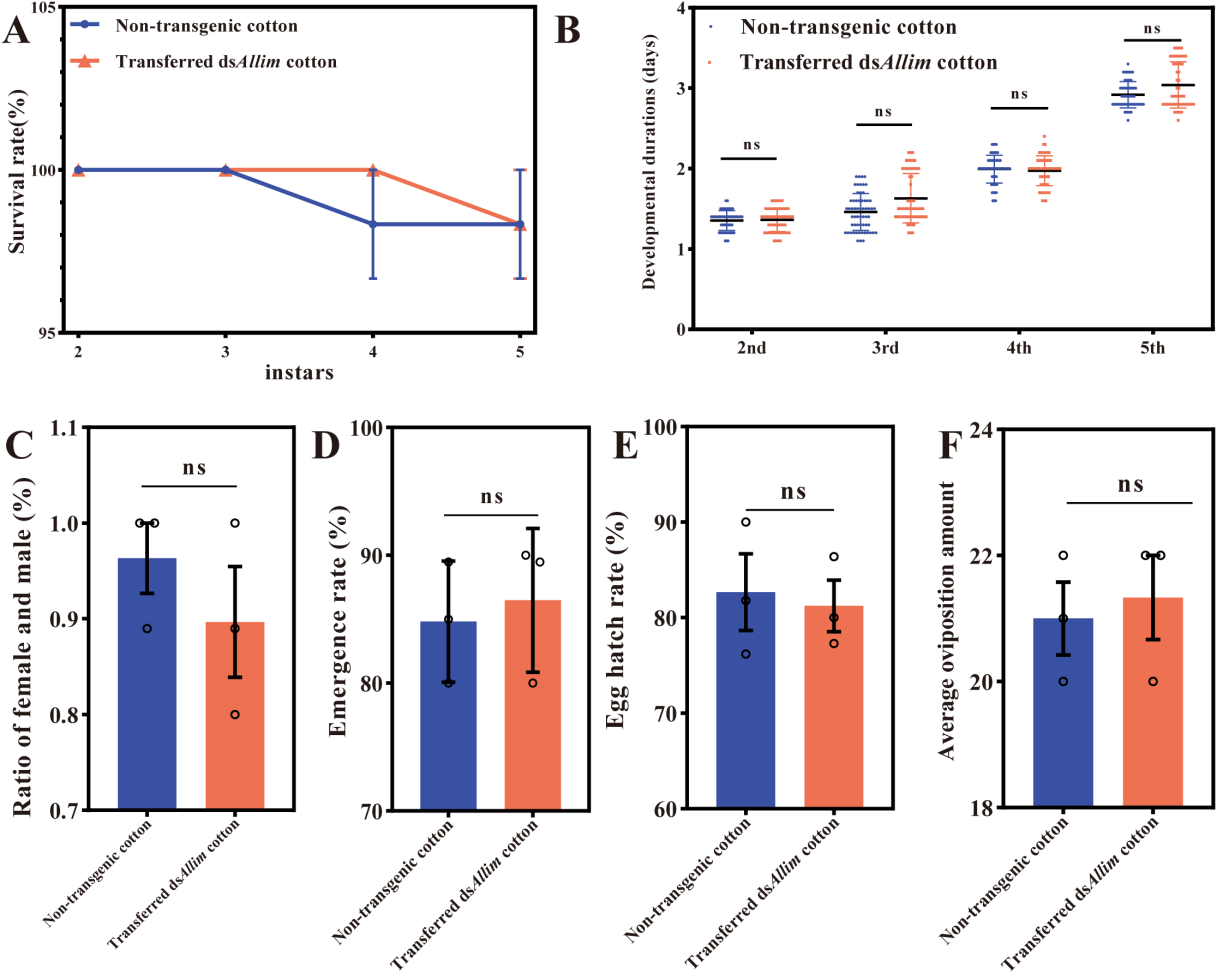
Life history parameters of *Orius similis* predators fed on aphids reared on dsAllim-transformed cotton and a non-transformed control cultivar. **(A)** Nymphal survival rate across instars. The horizontal axis represents the nymphal instar stages (2st to 5th). The vertical axis shows the cumulative survival rate (%). **(B)** Duration of each nymphal instar. The horizontal axis represents the nymphal instar stages. The vertical axis shows the mean duration (in days) spent in each stage. **(C)** Sex ratio (female: male). The vertical axis represents the ratio of females to males in the emerged adult population. **(D)** Final adult emergence rate. The vertical axis shows the percentage of nymphs that successfully emerged as adults. **(E)** Egg hatch rate. The vertical axis shows the percentage of laid eggs that successfully hatched. **(F)** Average oviposition per female. The vertical axis shows the mean number of eggs laid per female over a defined period. No statistically significant differences were observed in any of the measured parameters between the dsAllim and control groups (mean ± SD, n=3).

No significant difference was observed in the nymphs survival rate (**Figure 1A**), nymphs duration (**Figure 1B**), sex ratio (**Figure 1C**), emergence rate (**Figure 1D**), fecundity (**Figure 1E**) nor hatching rate (**Figure 1F**) of *O. similis*, when compared with the control treatment, indicating that there was no adverse effect of ds*Allim* cotton on the survival and development of *O. similis*.

### 3.3 Life-table parameters of *O. similis* were not affected by high dose of ds*Allim* in animal-feeding test

**Figure 2:**
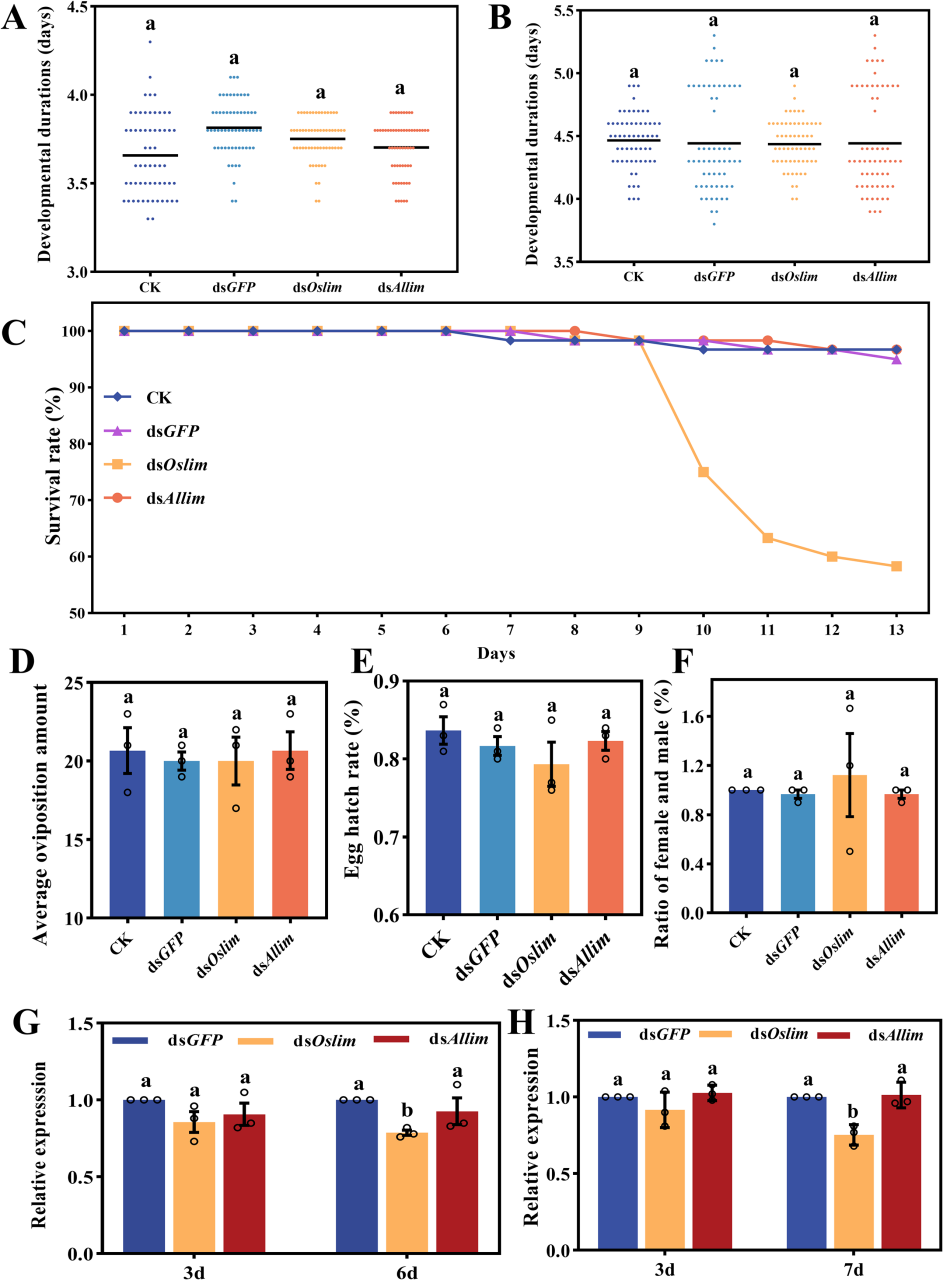
Evaluation of RNA interference of *Lim* gene in *O. similis*. **(A, B)** Duration of the nymphal stages: **(A)** fourth instar and **(B)** fifth instar. The vertical axis shows the developmental time in days. Data are compared between nymphs fed on a dsLim-treated artificial diet and those fed on a control diet (containing either dsGFP or no dsRNA) (n=20). **(C)** Cumulative survival rate of nymphs from the first instar to adult emergence. The vertical axis shows the survival percentage. **(D)** Average oviposition per female. The vertical axis shows the mean number of eggs laid per female over a defined period. **(E)** Egg hatch rate. The vertical axis shows the percentage of laid eggs that successfully hatched. **(F)** Adult sex ratio. The vertical axis shows the ratio of emerged females to males. **(G, H)** Relative expression levels of the OsLim gene: **(G)** in nymphs and **(H)** in adults. The vertical axis shows the normalized gene expression level (e.g., relative to actin). The horizontal axis distinguishes the dsLim treatment group from the control group(s).Values are mean ± SD from 3 replicates. Different lowercase letters indicate significant differences (P < 0.05, one-way ANOVA with Tukey's HSD test).

Even though an high dosage (the concentrations of each dsRNA were 440 ng/g which were 100× that expressed in cotton leaves of the ds*Allim* cotton) was applied in feeding test, no significant changes in the durations of the 4^th^ and 5^th^ instar stages was observed among dsRNAs (ds*GFP*, ds*Allim* and ds*Oslim*) and H_2_O treatments (**Figures 2A, B**). The larval survival rate in ds*Oslim* treatment showed a significant decrease at 9 d (beginning of pupation), and recorded about 42% of pupae mortality at 12 d. Besides, the larval survival rates were not significantly different among the ds*GFP*, ds*Allim* and H_2_O treatments (**Figure 2C**). Furthermore, the fecundity (**Figure 2D**), hatching rate (**Figure 2E**) and sex ratio (**Figure 2F**) for populations from all treatments, showed no significant difference. These results further showed that the ds*Allim* cotton had no adverse effect on the survival and development of *O. similis*.

### 3.4 Uptake efficiency of dsRNA in *O. similis*

There was no significant change in *Oslim* expression in both nymphs and adults of *O. similis* when they fed on all dsRNAs for 3 d (**Figures 2G, H**). However, significant silencing of the *Oslim* gene was observed in nymphs that fed for 6 d (**Figure 2G**) and adults that fed for 7 d (**Figure 2H**) on ds*Oslim.* However, feeding on both ds*Allim* and ds*GFP* did not induce significant change in *Oslim* expression in both nymphs and adults and all times (**Figures 2G, H**). The results showed that ds*Oslim* effectively suppressed *Oslim* expression in the animal-feeding assay. Also, the results may explain the mortality recorded in the ds*Oslim* treatment in above feeding test.

### 3.5 Off-target effects on non-homologous genes were induced by ds*Allim in O. similis*

**Figure 3:**
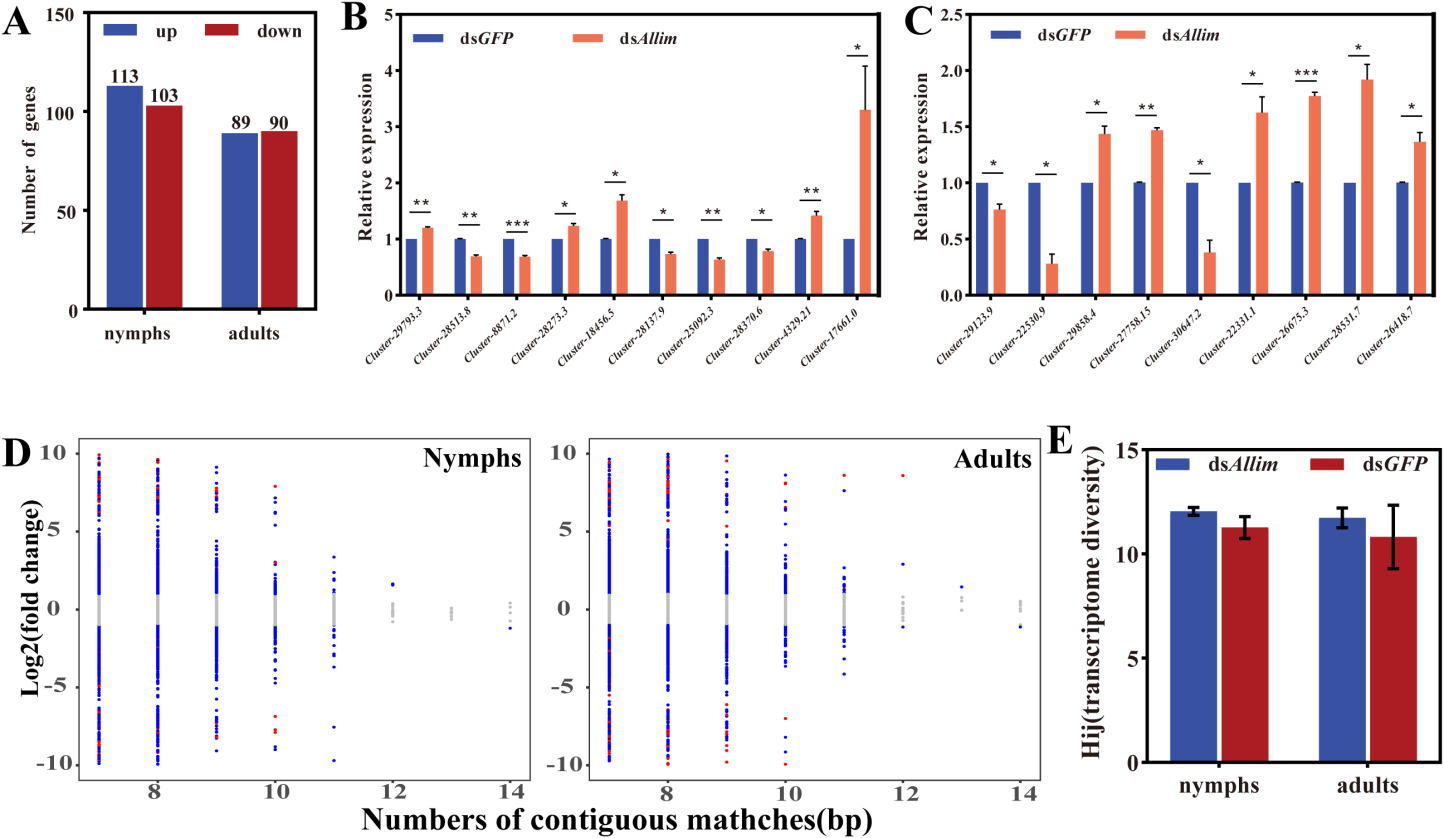
Transcriptome entropy for evaluating siRNA off-effects. Quantitative count of DEGs **(A)** for transcriptome sequencing and qRT-PCR validation for *O. similis* nymphs **(B)** and adults **(C)** Values are means SD (n = 3) (* P ≤ 0.05, ** P ≤ 0.01, *** P ≤≤ 0.001, Student’s t-test). **(D)** The number of base-pairs matched is linked with their fold changes for each continuous matched gene. The red points are genes that showed significant differential gene expression (|log_2_ (fold-change)≥1 and FDR ≤ 0.05). The blue points are genes that showed differential gene expression (|log_2_ (fold-change)≥1 and FDR ≥ 0.05). The gray points are genes that showed no differential expression (|log_2_ (fold-change)< 1). **(E)** The effect of siRNA feed on the Shannon transcriptome entropy. Error bar showed standard error (n=3).

The samples of *O. similis* which fed on ds*Allim* and ds*GFP* for 7 d in nymphs and 8 d in adults were sent for transcriptome sequencing, based on the dsRNA uptake efficiency results. Results showed that exposure to ds*Allim* induced down-regulation of 103 genes in nymphs and 90 genes in adults, compared to ds*GFP*, rather than by the *Oslim* gene family (**Figure 3A**). To validate the transcriptome data, we randomly selected 19 DEGs for qRT-PCR assays. All the expression profiles of the selected genes were consistent with the transcriptome results in both nymphs and adults (**Figures 3B, C**).

The number of base-pairs matched is linked with their fold changes for each continuous matched gene. The correlation between the number of matches and their fold changes showed that the highest expression changes were usually detected on genes with fewer consecutive matches (**Figure 3D**). This indicated that the causal relationships between the number of base matches and changes in their expression levels were diverse. The results showed that the Shannon entropy values did not change significantly following ingestion of ds*Allim* in both nymphs and adults (**Figure 3E**).

KEGG and GO enrichment analysis of DEGs showed that DEGs in ds*Allim*-treated nymphs showed no significant enrichment in the KEGG pathway associated with muscle development (**Supplementary Figure S1A**) but were significantly enriched in GO entries associated with epidermis development, molting cycle and cuticle development (**Supplementary Figure S2A**). In ds*Allim*-treated adults, the DEGs were more enriched in the focal adhesion KEGG pathway (**Supplementary Figure S1B**), but not in the muscle development-related GO entries (**Supplementary Figure S2B**). These results suggested that off-target effects were induced by ds*Allim* in *O. similis*.

### 3.6 The evaluation of off-target effects and transcriptome homeostasis

We classified the DEGs into 3 classes: (1) DEGs appearing in homologous target genes. We totally identified 8 homologues of the *Allim* gene in our local *O. similis* transcriptome, of which 7 or 5 were identified in larval or adult DEGs, respectively. However, all their expression profiles were not significantly affected. (2) DEGs involved in related KEGG enrichment pathways. Gene silencing within a functional network may trigger expression changes in other genes associated with the same KEGG pathway (level 1 pathway in **Table 1**), or other genes in pathways that interact with level 1 genes (level 2 pathway in **Table 1**) and so on (level 3 pathway in **Table 1**). Our results showed that exogenous ds*Allim* had an obvious effect on the related KEGG pathway genes in *O. similis*, which reached around 40% for nymphs and 30% for adult, even though it did not affect the expression of homologue genes (**Table 1**). (3) DEGs containing continuous matched base-pairs with dsRNA. Our analysis showed that the off-target effects did not correlate with the number of continuous matched base-pairs, and most of the DEGs had less than 10 continuous matched base-pairs. Furthermore, we also found that 96.43% ~ 100% *O. similis* genes containing continuous matched regions with ds*Allim* were not affected (**Table 2**). Correlation of the number of base-pairs matched and their fold changes for each continuous matched gene, showed that the highest expressive changes were usually detected on the gene with a lower number of continuous matches (**Figure 3D**). This indicated that importance should be attached to the diversity of the causality between the number of base-pairs matched and their expressive changes.

**Table 1:** The silencing of the related KEGG genes after ds*Allim* ingestions in *O. similis.*.

Transcriptome	KEGG pathway level	Total no. of genes	No. and percentage of up-regulated genes	No. and percentage of down-regulated genes	No. and percentage of non-significant-change genes
Nymph	Level 1	35	17 (48.57%)	18 (51.43%)	0 (0.00%)
	Level 2	280	47 (16.79%)	40 (14.29%)	193 (68.93%)
	Level 3	103	23 (22.33%)	22 (21.36%)	58 (56.31%)
	Total	418	87 (20.81%)	80 (19.14%)	251 (60.05%)
Adult	Level 1	65	15 (23.08%)	16 (24.62%)	34 (52.31%)
	Level 2	144	30 (20.83%)	25 (17.36%)	89 (61.81%)
	Level 3	198	27 (13.64%)	17 (8.59%)	154 (77.78%)
	Total	407	72 (17.69%)	58 (14.25%)	277 (68.06%)

**Table 2:** The silencing of the genes containing continuously matched regions with ds*Allim* in *O. similis* transcriptome.

No. of matched base pairs (bp)	Total no. of genes	No. and percentage of up-regulated genes	No. and percentage (%) of down-regulated genes	No. and percentage of non-significant-change genes
7	28744	71 (0.25%)	91 (0.32%)	28582 (99.44%)
8	14237	69 (0.48%)	58 (0.41%)	14110 (99.11%)
9	3847	18 (0.47%)	21 (0.55%)	3808 (98.99%)
10	943	5 (0.53%)	8 (0.85%)	930 (98.62%)
11	185	2 (1.08%)	0 (0%)	183 (98.92%)
12	28	1 (3.57%)	0 (0%)	27 (96.43%)
13	13	0 (0%)	0 (0%)	13 (100%)
14	8	0 (0%)	0 (0%)	8 (100%)
15	0	–	–	–

Shannon entropy of a transcriptome measures the overall variations in genes expressions, which can be used to judge the potential risks on fundamental biological processes that may emanate from exogenous dsRNA. Results showed that the values of the Shannon entropies were not altered by ingestion of ds*Allim* in both nymphs and adults (**Figure 3E**), which demonstrated that the ds*Allim* has no adverse effect on the transcriptome homeostasis of *O. similis*.

## 4 Discussion

The widespread use of IRGE crops, has generated many concerns of their negative and positive effects. Nowadays, the increasing application of IRGE crops and other novel biotechnologies in insect-pest management, rather than synthetic insecticides. The development of an effective and standardized risk evaluation framework of GE crops helps to prevent unintended consequences to public health, agriculture and conservation, but also facilitate the authentication and communication of the same biotechnology product among governmental regulators worldwide. Most released IRGE crops are transgenic *Bacillus thuringiensis* crops, and for which many reports about their environmental risks have been published so far. However, the emerging GE technologies that contributese to crop genetic improvement present novel technical and regulatory challenges, which require new approaches to address them. Among modern GE technologies, the development of RNAi crops is one of the novel biotechnologies. However, their potential environmental risks were remain incompletely understood (Yan et al., 2024). Moreover, a standardized framework for the environmental risk assessment of RNAi-based GMPs has not yet been established in China. The EFSA provides guidance on the risk assessment requirements for genetically modified plants, which includes molecular characterization, particular bioinformatic analysis and confirmation, as well as the evaluation of food and feed safety and dietary exposure associated with RNAi-based GMPs (EFSA et al., 2025).

Insects’ natural enemies play vital role in agrosystems (Kleijn et al., 2019). Previous risk assessment of IRGE crops on natural enemies mainly focused on judging the potential effects on their development and survival, of which the food-chain delivery system was always involved. The food-chain delivery toxic testing is analogous to the whole-food animal test that is generally applied in the regulatory testing of the potential risks IRGE crops may pose to human health. Arguably, the food-chain testing is the most appropriate system for assessing the route through which natural enemies are exposed, although other approaches should be considered before its application. In this study, the transmission of insecticidal dsRNA from an RNAi crop to a natural enemy was determined.

The animal-feeding test allows for the addition of high dose of the given insecticidal components into the risk assessment system, e.g. 10× or higher (100× in this study) than the field exposure dose, which presents a worst-case exposure scenario for judging safety. However, this requires the use of an effective artificial diet to produce accurate outcomes. Our results showed that, high dose of ds*Allim* had no adverse effect on the development and survival of *O. similis*, which indicated that the ds*Allim* is safe for the predator *O. similis*. Similarly, multiple studies have confirmed that dsRNAs targeting specific pests had no adverse effects on their natural enemies: ds*alphaCOP* for Brassicogethes aeneus was harmless to the parasitoid *Nasonia vitripennis* (Li et al., 2023); dsRNA targeting *Euschistus heros* posed no risk to *Telenomus podisi* (Castellanos et al., 2022); three dsRNAs against *Henosepilachna vigintioctopunctata* showed no organismal effects on *Propylea japonica* (Chen et al., 2023; Liu et al., 2023). Similar safety was observed in cases targeting *Nilaparvata lugens*, *Anoplophora glabripennis*, and *Dendroctonus frontalis*, with no harm to their respective natural enemies (Dang et al., 2021; Hollowell and Rieske, 2022; Li et al., 2022). However, insecticidal dsRNAs are not universally safe. For example, ds*vATPase-A* against *Diabrotica virgifera virgifera* significantly prolonged developmental time in *Adalia bipunctata* and reduced survival in *Coccinella septempunctata* (Haller et al., 2019; Pan et al., 2020). Therefore, the safety of RNAi crops toward natural enemies must be evaluated case by case. Additionally, homologous genes may function differently across species—suppression of Allim in *A. lucorum* caused molting failure and death (Liang et al., 2021), whereas high doses of ds*Oslim* had no such effect on *O. similis*.

RNAi causes target effects on organismal transcriptomes (Jackson et al., 2003), which generates public concerns about the potential adverse effects of RNAi crops on non-target species (Lundgren and Duan, 2013). The omics technologies have been applied to evaluate these off-target effects in many insects Wu et al., 2022; Chen et al., 2023). Here, our transcriptomic analysis also identified ~200 unintended DEGs induced by ds*Allim* in nymphs and adults of *O. similis*. We acknowledge that our enrichment analysis did not reveal any muscle-related pathways among the differentially expressed genes (DEGs). In this context, the observed enrichment of off-target DEGs in biological processes such as epidermis development and the molting cycle is particularly noteworthy. As these specific, non-target pathways were unexpectedly perturbed following *AlLIM* knockdown, we propose that this phenomenon may represent indirect effects triggered by the disruption of this key regulatory gene. The potential for RNAi constructs to induce such unintended transcriptomic changes warrants further investigation in future ecological risk assessments. Furthermore, our data showed that the off-target effects did not correlate with the number of continuously matched base-pairs. The number of continuous matched base-pairs between gene and exogenous dsRNA was as low as 7 to trigger RNAi, which was consistent with our previous study (Wu et al., 2022), but different with others (Chen et al., 2021, 2023). This indicated how flexible the transcriptome may be to exogenous dsRNA. Therefore, the unintended regulations of transcriptomes should not be ignored in the safety evaluations of RNAi crops on NTOs. However, such intended nor unintended transcriptomic changes do not fully indicate potential risks. The application of the Shannon entropy can enable the overall judgment of transcriptome stability (Wu et al., 2022) and should be considered in future studies and regulatory decisions, to gain public confidence.

## 5 Conclusion

We proposed a risk evaluation framework for RNAi crop on insects’ natural enemies, which could consist of (1) a food-chain delivery testing, which should explore the situation on the ground. (2) the assessment of exposure routes for insecticidal dsRNAs, which should be completed before any other evaluation experiments. The absence of such exposures abolishes the need for evaluation. (3) Animal-feeding testing, which should reveal the worst-case effects. (4) an off-target effects analysis, which should show potential unintended silencing effects. (5) a judgment of transcriptomic stability, which would unveil the overall transcriptome balance of the evaluated organism. Besides, our study also illustrated that the ds*Allim* cotton had no adverse effect on *O. similis* at both developmental and transcriptomic levels. Our findings, together with other previous studies, also demonstrated that the safety evaluation of RNAi crops on NTOs should be a case-by-case study, especially for the off-target effect analysis. This will require further studies to unravel the underlying mechanisms of off-targets by RNAi.

## Data Availability

The original contributions presented in the study are included in the article/**Supplementary Material**. Further inquiries can be directed to the corresponding authors. The raw datasets used for RNA-seq were deposited into the China National Center for Bioinformation (CNCB) SRA database under the accession numbers PRJCA043540.

## Supplementary material

Supplementary Figure 1 The KEGG analysis of transcriptomes.

Supplementary Figure 2 The GO analysis of transcriptomes.

Supplementary Table 1 Primers used for the synthesis of ds*Allim* and ds*Oslim.*

Supplementary Table 2 Primers used for qRT-PCR.

Supplementary Table 3 Primers used for PCR validation of DEGs for *O. similis* RNA-seq.

Supplementary Table 4 Primers used for qRT-PCR of DEGs for *O. similis* RNA-seq
