# Supplementary figures and images for: Using transcriptome analysis to evaluate the impact of dsAllim cotton on non-target organism O. similis
Source: Front Plant Sci. 2026 Jan 27;16:1720420. doi: 10.3389/fpls.2025.1720420 (PMC12886505; doi:10.3389/fpls.2025.1720420)

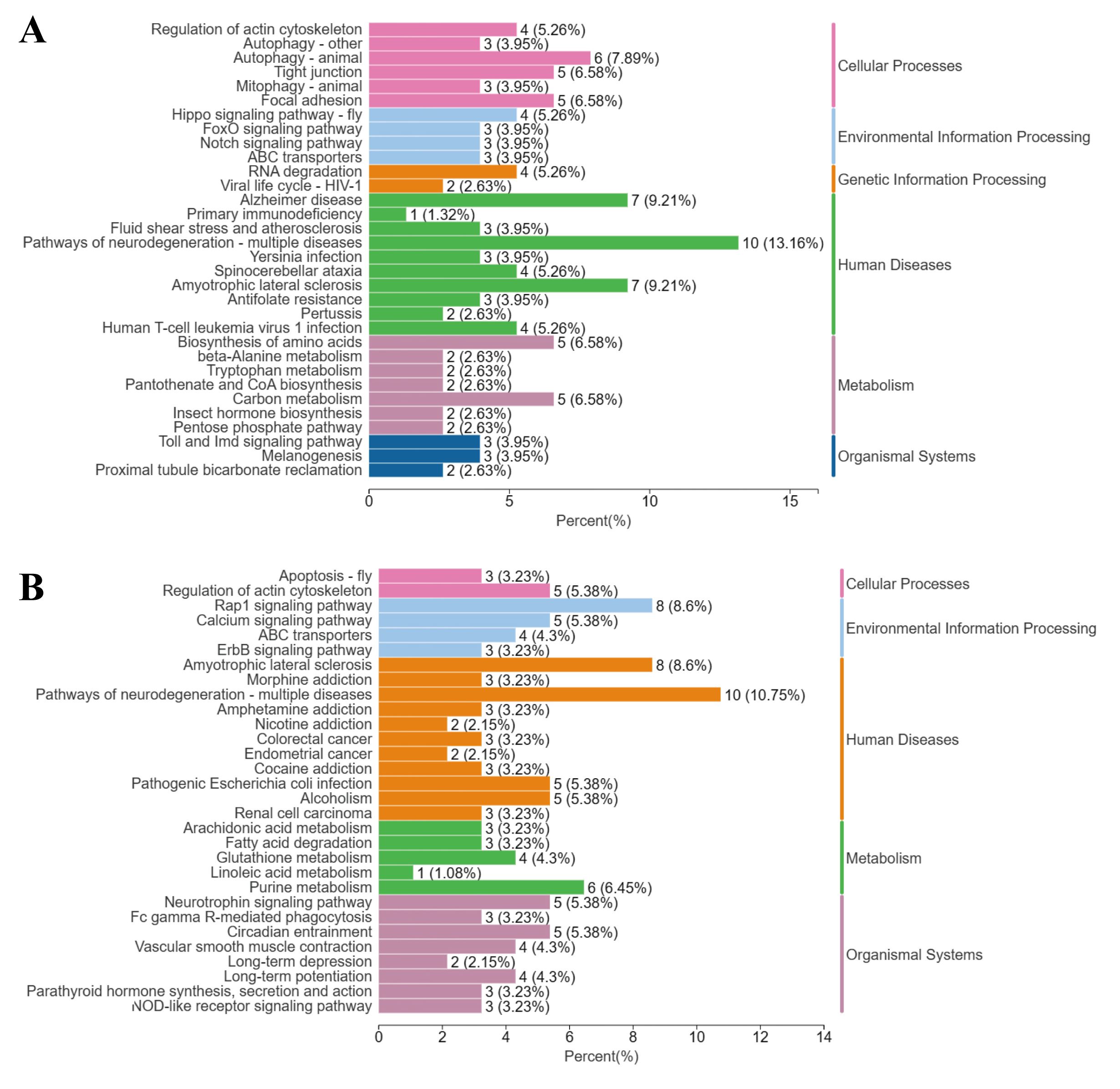

Supplement: Supplementary Figure 1 — The KEGG analysis of transcriptomes. [file Image1.tif]

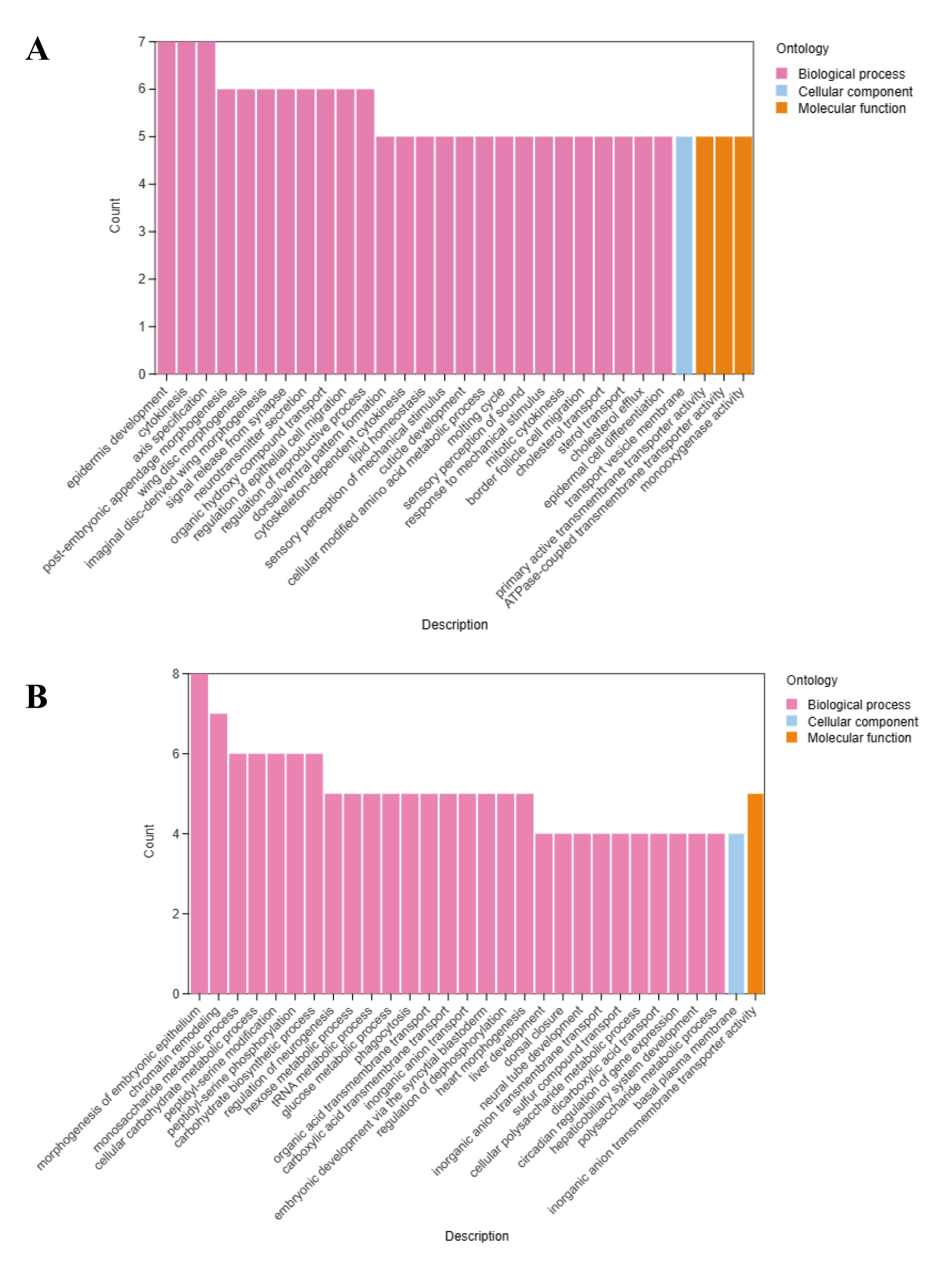

Supplement: Supplementary Figure 2 — The GO analysis of transcriptomes. [file Image2.tif]
