# Supplementary material for: Using transcriptome analysis to evaluate the impact of dsAllim cotton on non-target organism O. similis
Source: Front Plant Sci. 2026 Jan 27;16:1720420. doi: 10.3389/fpls.2025.1720420 (PMC12886505; doi:10.3389/fpls.2025.1720420)
Supplement: Supplementary Table 1 — Primers used for the synthesis of dsAllim and dsOslim. [file Table1.docx]

**Table S1** Primers used for the synthesis of ds*Allim* and ds*Oslim*

| Primer Name | Primer sequence (5’ - 3’) |
| --- | --- |
| ds*Allim*-F | taatacgactcactataggCTGGAATCGTGCCGTGTCT |
| dsA*llim*-R | taatacgactcactataggCGCTGGCTGTCTCAGTATGG |
| ds*Oslim*-F | taatacgactcactataggTGCCTCAGCATGGACAAG |
| ds*Oslim*-R | taatacgactcactataggTTTATGCATGCAGTATCCACG |
| ds*GFP*-F | taatacgactcactataggTGGTCCCAATTCTCGTGGAAC |
| ds*GFP*-R | taatacgactcactatagg CTTGAAGTTGACCTTGATGCC |

**Table S2** Primers used for qRT-PCR

| Primer Name | Primer sequence (5’ - 3’) |
| --- | --- |
| q*Oslim*-F | TCAGTTTATGCGGCAGAGGAAC |
| q*Oslim*-R | TAAAGTTCACCTTCGTGCTCGG |
| *β-actin*-F | ACCGCTGCTGCTTCCAC |
| *β-actin*-R | ACCGCAAGATTCCATACCC |

**Table S3** Primers used for PCR validation of DEGs for *O. similis* RNA-seq

| **Gene** | **Primer-F (5’ - 3’)** | | **Primer-R (5’ - 3’)** | |
| --- | --- | --- | --- | --- |
| *Cluster-29793.3* | | TCCGATTTTAATTGATGGTGGC | | ATCAACTAAAGCAAGGGCTTCAA |
| *Cluster-28513.8* | | CATTTGCATGAGACATGGACCC | | CGTCGGTCCTAACCCTTTTATTG |
| *Cluster-8871.2* | | CACCGAAACAACGAACGTCTAGC | | CTTTCGGAAATTTGCCACATCG |
| *Cluster-28273.3* | | ATCTCGCAGAGTTGGCTCTAGCA | | TGGACGTGATTTTCGCCTCCTA |
| *Cluster-18456.5* | | GGTTTTCCAGACGAAGCTTTTCC | | CCATCAAACGATTTTCCGGG |
| *Cluster-28137.9* | | ATGAGTGACATGTCAGCCCCA | | CATGTGAGAATCCGTTGGTCAA |
| *Cluster-25092.3* | | ATGGAAAGGCGCCTAAGCACTA | | ACTAAATTTGGCACCGTCATCGC |
| *Cluster-28370.6* | | GGTCATTTAACAATGGCCCCGA | | TTACTTTTCAGGCTGCGGGAG |
| *Cluster-4329.21* | | GGTCGCTAAGATTTTCGTTGTTG | | CATAAGCGACCGGAGCGTGA |
| *Cluster-17661.0* | | GGAATTCGAAGACAAGATTGGC | | TCACCAAGAGCTTTGCGTTTCT |
| *Cluster-29123.9-1* | | ATGACCAACTCATGGACTTTGACAA | | ACTTCCATTATGTCAGGAATCGTGG |
| *Cluster-29123.9-2* | | GCGGCTCTTATGTGCTATAATACAG | | GACCATTCCAGAAGGTTGCTGT |
| *Cluster-29123.9-3* | | TATTCGGGAATAAGCCGAAAAG | | TTACCTGCTTAATGTCGTCGACG |
| *Cluster-22530.9* | | GAAATGCAATACCCGAAAATCTC | | CTTAACTTTCTTCAGCTTGATCGTT |
| *Cluster-29858.4* | | ATGGGTAACGTCCGTTCGGAAGAT | | AATGGTTTTGTTGCAGCAGGCC |
| *Cluster-27758.15* | | GAACAAAGCAGAAGAGACGATTTAG | | ATTGCCCCTCCTGAAACTGAG |
| *Cluster-30647.2* | | CACCGAAAACGAGCATCACAGG | | GCTGATGCCTTTTTCTTCGAAGAG |
| *Cluster-22331.1* | | ATAATGGCAGCGATGGAGAGAGT | | AGAATTAATAAAATCGGCGACGAT |
| *Cluster-26675.3* | | CTCCGGAAGCACCTGTTTTTGAC | | CGCAGTCGGTATATTGGCTTTTC |
| *Cluster-28531.7* | | ACCCACGAAGAAGCTCCATCAAC | | CACCGTCTTCTCCTACTCACAGCC |
| *Cluster-26418.7* | | ATGGTGAAAAACATTGAGCCATGG | | GGAGGGCTTCCAAAGAGTCTTCT |

**Table S4** Primers used for qRT-PCR of DEGs for *O. similis* RNA-seq

| Gene | Primer-F (5’ - 3’) | Primer-R (5’ - 3’) |
| --- | --- | --- |
| *Cluster-28513.8* | AATTTTGGGGCGACGTCACTTATTT | GCGTTGCAATGGTTTTGAATGTCTT |
| *Cluster-8871.2* | CGAAATCCCCGAAGTCCTTATTTGG | TTTTTCCCTATCTTTGGCGTCGTTC |
| *Cluster-28273.3* | CGAAAAGAGACGATGACGCAGATAC | TGAGTGGCTTTTGATTTCGTTGAGG |
| *Cluster-18456.5* | AAAGAGGAGAGCTAAACTCGAGCAA | TGTTTGTTATGAAGCCACTTCCAGG |
| *Cluster-28137.9* | GAAGAATTTTGCCTCCCGACAAAGA | TGTGAGAATCCGTTGGTCAATTTCG |
| *Cluster-25092.3* | AGAAAGAGTCAACTGGGATGGACAA | CCTTTCATGGCGGACTTTTTCTCTT |
| *Cluster-28370.6* | TAGGCTATTCAAGTCCTACGCTGAC | AAGTAAACCGGGAACTAACTGACGA |
| *Cluster-4329.21* | AAAACCGTCGTTGCTGACGAATAC | CAGGTTCGACTAAGGAGTAGCTTCC |
| *Cluster-17661.0* | ATTTGCGTGTGAAAACAAACCGAAC | TTTCATTTCTCGCCTTTCTTTCGCT |
| *Cluster-29123.9* | ACAGAAGGGACTCGACCAACATAAA | TAGAATGGTTTTCACAAAGACCGCC |
| *Cluster-22530.9* | TAGAGGAACTCAAACCAGAAGAGCC | TTTTCGGGTATTGCATTTCAGGAGG |
| *Cluster-29858.4* | CCTCCGTCAATTCTTACATCTTCGC | GTCGTTGGAAGTTAAAAGAGGGGTG |
| *Cluster-27758.15* | GCCGCAACGAAACGTCAAAAATATG | GAATAGTCGGGCCTTTCTTCAAACC |
| *Cluster-30647.2* | AGATGCTATGCATGAAATGGGAAGC | CCATAGACCAGTCAGAACCACTGAA |
| *Cluster-22331.1* | CAACTTCCCATCGGGCAATATCATC | CTTTCGGGAATGAGTGGTAAACAGG |
| *Cluster-26675.3* | CGAGCGTACCATTCAGGATTTAACC | AAATCAACTCGTCGTGGGAGAAAAC |
| *Cluster-28531.7* | ACAACGACACTTCCACCTCATTTTC | TGGTCCAGAAGTCGAGGGTATTTTT |
| *Cluster-26418.7* | CGAAGAAGAAGAGAAAACGACGGAG | GCACATTAAACGGACTGCATTTGTC |
